# Inflatable penile prosthesis placement after prior transcorporal artificial urinary sphincter placement: A case report

**DOI:** 10.1016/j.eucr.2022.102310

**Published:** 2022-12-29

**Authors:** Leah Ashton, Brad Erickson, Amy Pearlman

**Affiliations:** University of Iowa Hospitals and Clinics, Department of Urology, United States

**Keywords:** Inflatable penile prosthesis, Artificial urinary sphincter, Transcorporal AUS, Prostatectomy complications, Case report, SUI, stress urinary incontinence, IPP, inflatable penile prosthesis, AUS, artificial urinary sphincter, RTE, rear tip extenders

## Abstract

Stress urinary incontinence and erectile dysfunction often coexist in men surgically treated for prostate cancer. Despite many men having both an artificial urinary sphincter and inflatable penile prosthesis to treat these conditions, there is limited information in the literature to guide surgeons when it comes to placing both devices.

We recommend obtaining direct exposure of proximal crura to allow for complete dilation of corporal spaces for proper prosthetic placement. Further dissection via penoscrotal incision or perineal counter-incision can be utilized. Surgeons should consider dorsal lithotomy position at time of IPP placement to allow for perineal exposure.

## 1 Introduction

Radical prostatectomy and pelvic radiation often lead to erectile dysfunction (ED) and stress urinary incontinence (SUI). ED affects up to 85% of men after radical prostatectomy,^1^ with the inflatable penile prosthesis (IPP) being the most definitive treatment to restore function, whereas the artificial urinary sphincter (AUS) is the most effective long-term treatment for men with SUI.^2^

Single and dual incision techniques have been developed for simultaneous placement of IPP and AUS. However, more often, these procedures are done asynchronously as most men elect incontinence management first. In cases of urethral fragility where a transcorporal AUS cuff is required, subsequent IPP placement can be challenging, and there is limited data on how to best place IPPs to minimize complications given AUS cuff already occupies the corporal space. We present a case of IPP placement in the setting of previously placed AUS with a resulting complication and subsequent surgical repair.

## 2 Case presentation

64-year-old male s/p radical retropubic prostatectomy and adjuvant radiation therapy for Gleason 9 prostate adenocarcinoma presents with interest in IPP.

The patient had undergone three prior surgeries for post-prostatectomy SUI: an initial urethral sling, followed by mid-bulbar AUS cuff and ultimate transcorporal cuff via a perineal incision. At presentation, he was satisfied with his mild SUI.

Patient was placed in supine position with AUS deactivated and foley catheter in place. After penoscrotal incision made for exposure of corporal bodies, Brooks dilators were used for proximal dilation of corporotomies. Notably, resistance was met early, and the surgeon made the decision not to attempt dilation proximal to the AUS cuff. The total corporal measurement was 13.5 cm bilaterally, and a 12 cm AMS LGX implant with 1.5 cm rear tip extenders (RTE), was placed without difficulty. A sub-rectus concealed reservoir was placed via a counter incision. Intraoperative cycling of the IPP revealed excellent functional and cosmetic results.

The implant was deflated and catheter removed on post op day 1. The AUS remained deactivated until his one-week post-operative visit, at which time the patient demonstrated the ability to use his AUS pump. IPP wounds were healing well.

**Fig. 1:**
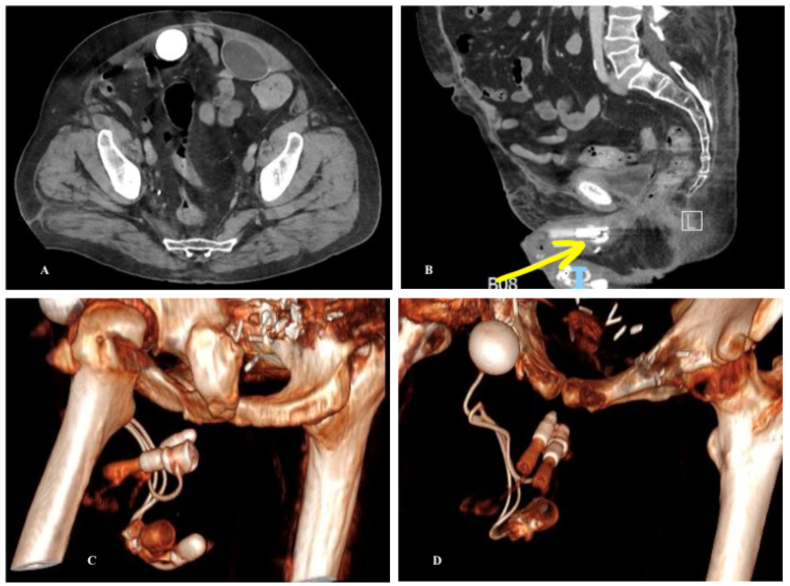
**a.** Post operative CT demonstrating prior AUS reservoir (on patient's right; filled with contrast) and recently placed sub-rectus IPP reservoir (on patient's left). **b.** Post operative CT demonstrating proximal end of the left IPP cylinder through the lumen of the transcorporal AUS cuff. **c and d.** 3D reconstruction demonstrating proximal end of the left IPP cylinder through the lumen of the transcorporal AUS cuff**.**

The patient presented for a wound check at 3 weeks, complaining of lower abdominal pain, dysuria, constipation, and worsening urinary incontinence despite his activated AUS. Concern for urethral erosion prompted a cystoscopy which showed no device erosion. A CT scan was obtained which demonstrated the RTE within the AUS (Fig. 1A–D).

Given the likelihood of impending urethral erosion, patient was taken back to the operating room semi-emergently for penile and perineal exploration. The patient was placed in dorsal lithotomy, and a vertical midline perineal incision was made to expose bilateral proximal crura lateral to the existing transcorporal cuff. Bilateral corporotomies were then performed without exposing the cuff, and the RTE were visualized. After ensuring that the cuff had not been damaged and the urethra was intact, the proximal crura were then dilated proximal to the AUS cuff, of which the dorsal aspect was not visible. The RTEs were changed from 1.5 cm to 4 cm and directed away from the AUS. Absorbable suture was used to roughly close space between the left sided proximal corporotomy and AUS cuff at the location where the left cylinder had passed through the transcorporal cuff. Post-operative cystoscopy confirmed no urethral injury and cycling of his AUS under direct vision demonstrated complete opening and urethral cuff coaptation.

Six weeks following his IPP revision, patient is satisfied with his current continence and has been cleared to use his IPP.

## 3 Discussion and conclusions

ED and SUI are common complications after radical prostatectomy and radiation therapy, yet minimal information is documented in the literature regarding how to best place IPPs in patients with existing transcorporal AUS cuffs occupying the corporal space.

For patients with IPPs in place with SUI, subsequent transcorporal AUS placement has been described. In patients with subcuff atrophy, AUS cuff erosion, radiation history, and/or previous AUS revisions, transcorporal AUS may be placed using a 6-ply acellular graft via perineal incision. This graft may provide adequate separation between AUS and IPP components and hemostasis. The graft traverses the transcorporal dissection plane and is sutured to the lateral edges of the corporotomies and the AUS cuff is placed between the urethra and graft.^3^

However, only a single article describes IPP placement after transcorporal AUS placement. Amongst the three cases described, one patient had AUS implantation via perineal approach followed by later IPP placement via penoscrotal approach. The other 2 cases had initial penoscrotal transcorporal AUS placement followed by infrapubic IPP placement. All three patients reported satisfactory intercourse, no device infection or erosion, and consistent level of continence following IPP placement.^4^

At time of initial IPP placement, our patient was positioned supine. When dilating the proximal crura, resistance was met early and dilation was not attempted proximal to the AUS cuff. In this circumstance, the proximal crura should be exposed to allow for direct visualization and careful dilation of entire corporal space bilaterally so the proximal aspect of the IPP cylinders do not put the AUS cuff at risk of injury. To best expose the proximal crura via the existing penoscrotal incision, the dissection could have been extended further proximally, as has been described for penoscrotal placement of an AUS cuff.^5^ If still insufficient exposure through the existing penoscrotal incision, authors recommend making an additional perineal incision to expose bilateral proximal crura and to allow for direct access to the existing transcorporal cuff. Patient positioning in dorsal lithotomy, rather than supine, allows for sterile field maintenance and efficient exposure of anatomy for complex cases that may necessitate a perineal counter-incision.

Future studies could focus on long term outcomes of this patient population, as this was a limitation of our study.

Our experience adds to prosthetic literature on operative technique in complex patients with both ED and SUI. Existing literature on penoscrotal or infrapubic incision suggest both as viable options. Direct exposure to proximal crura and complete dilation of corporal spaces are critical to ensure proper and safe prosthetic placement.

## Ethics approval and consent

Not applicable.

## Consent for publication

Written informed consent was obtained from the patient for publication of this case report and any accompanying images.

## Availability of data and materials

Not applicable.

## Funding

Amy Pearlman and Bradley Erickson are consultants for and receive grant funding from Boston Scientific (but did not fund this publication)

## Authors' contributions

LA analyzed the patient data and wrote the draft, AP and BE provided expertise and edited the manuscript. All authors read and approved the final manuscript.

## Declaration of competing interest

Amy Pearlman and Bradley Erickson are consultants for and receive grant funding from Boston Scientific.
